# Trends, Disparities, and Forecasts in Co-Recorded Pancreatic Cancer and Cerebrovascular Disease Mortality in the United States, 1999–2024

**DOI:** 10.3390/cancers18152431

**Published:** 2026-07-28

**Authors:** Arkadeep Dhali, Jyotirmoy Biswas, Ali Shan Hafeez, Sajjad Ahmed Khan, Dushyant Singh Dahiya, Saikat Mandal

**Affiliations:** 1 Academic Unit of Gastroenterology, Sheffield Teaching Hospitals NHS Foundation Trust, Sheffield S10 2JF, UK; 2 School of Medicine, Dentistry and Biomedical Sciences, Queen’s University, Belfast BT9 7BL, UK; 3 Department of Medicine, Barasat Government Medical College and Hospital, Barasat 700124, India; 4 Department of General Medicine, CMH Multan Institute of Medical Science, Multan 60000, Pakistan; 5 Department of Internal Medicine, Birat Medical College Teaching Hospital, Morang 56613, Nepal; 6 Division of Gastroenterology, Hepatology & Motility, The University of Kansas School of Medicine, Kansas City, MO 66160, USA; 7 School of Medicine, University of Nottingham, Nottingham NG7 2UH, UK

**Keywords:** pancreatic cancer, cerebrovascular disease, mortality, health disparities, CDC WONDER

## Abstract

**Background:** The population mortality burden associated with co-recorded pancreatic cancer and cerebrovascular disease (i.e., deaths in which both conditions are listed on the same death certificate) in the United States remains poorly characterized. We evaluated temporal, demographic, geographic, urbanization, and forecasted mortality trends. **Methods:** This retrospective population-based study used CDC WONDER Multiple Cause of Death data for adults aged 25 years or older from 1999 to 2024. Individuals younger than 25 years were excluded because pancreatic cancer mortality is extremely uncommon in this age group, resulting in sparse counts and unstable age-adjusted mortality estimates. Deaths were included when both conditions were listed as underlying or contributing causes. Age-adjusted mortality rates (AAMRs) were analysed using Joinpoint regression, and autoregressive integrated moving average models generated 10-year forecasts. **Results:** Overall, 22,085 deaths were identified. Annual deaths increased from 591 in 1999 to 1630 in 2024 while the AAMR increased from 0.32 to 0.56 per 100,000. Rates were stable from 1999 to 2013, then increased significantly through 2024 (annual percentage change, 6.26%; 95% confidence interval, 5.11–8.25). Mortality was higher among males, reaching 0.65 per 100,000 in 2024, and highest among non-Hispanic Black or African American individuals, at 0.87 per 100,000. Full-period AAPCs were significantly positive across all Census regions, with the highest 2024 AAMR in the Midwest at 0.57 per 100,000. Metropolitan and nonmetropolitan rates reached 0.45 per 100,000 by 2020. The overall AAMR was projected to reach 0.76 per 100,000 by 2034. **Conclusions:** US co-recorded pancreatic cancer and cerebrovascular disease mortality increased substantially after 2013, with persistent sex, racial, and geographic disparities.

## Simple Summary

Pancreatic cancer and cerebrovascular disease (conditions such as stroke) have each been extensively studied individually, but little is known about how often they are recorded together on death certificates and whether this pattern is changing over time. Using United States mortality data from 1999 to 2024, we found that deaths listing both pancreatic cancer and cerebrovascular disease increased sharply after 2013, with the increase most pronounced among men and non-Hispanic Black or African American individuals. These findings describe a population-level mortality pattern, rather than an individual patient’s risk of stroke after a pancreatic cancer diagnosis, and further studies using linked clinical data are needed to clarify the reasons behind this trend.

## 1. Introduction

Pancreatic cancer remains among the most lethal gastrointestinal malignancies. Population-based analyses show that its incidence and mortality have increased in many countries, including the United States, despite modest improvements in survival [1,2]. Beyond tumour progression, thrombotic complications represent an additional clinical burden. In a SEER-Medicare cohort, newly diagnosed cancer was associated with increased short-term stroke risk, with pancreatic cancer among the tumour types showing the greatest excess risk [3]. A Taiwanese nationwide cohort likewise found increased incidences of both ischaemic and haemorrhagic stroke among patients with pancreatic cancer, particularly during the first six months after diagnosis [4]. Related US data demonstrated a substantially elevated six-month risk of arterial thromboembolism, including ischaemic stroke, after cancer diagnosis, with risk increasing according to cancer stage [5].

These observations support an important epidemiological intersection between pancreatic malignancy and cerebrovascular disease. However, existing investigations have primarily examined incident stroke or thromboembolism after cancer diagnosis. The long-term population-level mortality burden among individuals in whom pancreatic cancer and cerebrovascular disease coexist on death certificates, and its variation by sex, race/ethnicity, geography, and urbanization, remains poorly characterized. We use the term ‘co-recorded mortality’ to denote deaths where both conditions appear on the same certificate as either the underlying or a contributing cause. This is an observational metric and does not imply a causal relationship or individual complication risk.

Accordingly, this study evaluated US mortality involving both pancreatic cancer and cerebrovascular disease from 1999 through 2024 using CDC WONDER. We examined temporal trends in age-adjusted mortality rates overall and across demographic and geographic subgroups, assessed state-level variation, and projected future mortality using autoregressive integrated moving average models. Characterising these trends may identify disproportionately affected populations and provide a descriptive epidemiological foundation for future patient-level investigations.

## 2. Methods

### 2.1. Study Design and Data Source

This retrospective, population-based study used publicly available mortality data from the Centers for Disease Control and Prevention Wide-ranging Online Data for Epidemiologic Research database. Death records were obtained from the CDC WONDER Multiple Cause of Death records, which include one underlying cause of death and up to 20 contributing causes reported on U.S. death certificates (CDC Wonder Database Version 3.28.2) [6]. The study followed STROBE reporting guidance [7].

### 2.2. Case Definition

Deaths were included when both pancreatic cancer and cerebrovascular disease were listed on the death certificate as either underlying or contributing causes of death. Pancreatic cancer was identified using ICD-10 codes C25.0, C25.1, C25.2, C25.3, C25.4, C25.7, C25.8, and C25.9. Cerebrovascular disease was identified using ICD-10 codes I60–I69. Cases were identified using the CDC WONDER Multiple Cause of Death query interface, which searches the underlying cause-of-death field together with the contributing-cause fields for the specified ICD-10 codes. CDC WONDER tabulates unique death records, so each death contributed once to the resulting count. Where CDC WONDER marked an annual rate as ‘Unreliable’ because of a small numerator, that estimate was not used for subgroup trend interpretation. Data for 2024 reflect final mortality data as of the date of data extraction (6 June 2026). Either pancreatic cancer or cerebrovascular disease could be recorded as the underlying cause of death, with the other condition listed as a contributing cause. Duplicate records were not possible because each CDC WONDER record represents a unique death certificate.

To provide disease-specific context for the co-recorded mortality trend, two additional pairs of CDC WONDER queries were conducted, restricted to pancreatic cancer (ICD-10 codes C25.0–C25.9) and cerebrovascular disease (ICD-10 codes I60–I69) as the underlying cause of death, using the same age restriction (25 years and older) and the same two-database, non-overlapping calendar structure as the co-recorded series: the Multiple Cause of Death, 1999–2020 database for 1999–2020, and the Multiple Cause of Death, 2018–2024, Single Race database for 2021–2024. For each year, the ratio of co-recorded deaths to single-disease underlying-cause deaths was calculated as (co-recorded deaths ÷ single-disease underlying-cause deaths) × 100, expressed as co-recorded deaths per 100 underlying-cause deaths of the comparator condition. Because the co-recorded case definition counts either condition as a contributing cause, whereas the comparator queries count each condition only as the underlying cause, the co-recorded numerator is not necessarily a subset of either single-disease denominator; these ratios are therefore reported as descriptive context for the relative frequency of co-recording, rather than as a true proportion of all deaths in which each disease was mentioned.

The study period was January 1999 through December 2024. Urbanization analyses were limited to 1999 through 2020 because urban–rural classification variables were available only through 2020 in the CDC WONDER database. Accordingly, urbanization-stratified trends were estimated from 1999–2020 data, and their forecasts were limited to 2030.

### 2.3. Study Variables and Outcomes

The study population included U.S. decedents aged 25 years and older. This lower age threshold was selected because pancreatic cancer mortality is rare before age 25 and younger age groups would contribute very few co-recorded deaths, resulting in unstable estimates. Mortality was analyzed by year, sex, race/ethnicity, U.S. Census region, state, and urbanization status. Race/ethnicity was categorized as non-Hispanic White, non-Hispanic Black or African American, and Hispanic or Latino. American Indian or Alaska Native and Asian or Pacific Islander subgroups were not analyzed separately because annual estimates for these groups were frequently suppressed or flagged as unreliable by CDC WONDER because of small death counts, precluding stable year-by-year trend analysis. Census regions were defined as Northeast, Midwest, South, and West. Urbanization was classified using the National Center for Health Statistics Urban–Rural Classification Scheme and grouped as metropolitan or nonmetropolitan. Urbanization analyses were restricted to 1999–2020 because NCHS urbanization classifications were not available in the 2021–2024 provisional mortality data.

The primary outcome was the annual age-adjusted mortality rate per 100,000 population for deaths involving both pancreatic cancer and cerebrovascular disease. Rates were standardized to the 2000 U.S. standard population using the direct method [8]. Secondary outcomes included crude mortality rates, death counts, subgroup-specific mortality patterns, state-level variation, and projected mortality rates.

### 2.4. Statistical Analysis

Temporal trends in age-adjusted mortality rates were analyzed using the Joinpoint Regression Program, version 6.0.1, from the National Cancer Institute. Models were fitted using Grid Search, with a minimum of two observations required before the first and after the last joinpoint and between adjacent joinpoints, no additional grid points between observed years, and a maximum of four joinpoints. The number of joinpoints was selected using Weighted Bayesian Information Criterion rather than permutation testing. The rates were weighted using their standard errors. Confidence intervals for APC and AAPC were constructed using the empirical quantile method with 5001 resamples. Joinpoint regression estimated annual percent change (APC) and average annual percent change (AAPC) with 95% confidence intervals. Because 12 series were modeled, some subgroup-specific inflection points and segment estimates may reflect sampling variability, particularly where confidence intervals were wide.

Forecasting analyses were performed using autoregressive integrated moving average (ARIMA) models in R (forecast package) (R version 4.6.1). Annual age-adjusted mortality rate (AAMR) data for each stratum were converted to annual time-series objects (ts, frequency = 1, reflecting non-seasonal annual data) and fitted separately using the auto.arima function with default settings, which applies the Hyndman–Khandakar stepwise algorithm to select the optimal model by minimizing the corrected Akaike Information Criterion (AICc) [9]. No external regressors or data transformations were specified. The selected model order for each series was extracted using arimaorder, and the corresponding AIC and Bayesian Information Criterion (BIC) values were recorded. Ten-year forecasts (forecast(fit, h = 10)) were generated, yielding point estimates and 95% prediction intervals. Model adequacy was assessed using the Ljung–Box test at lag 10, and forecasting performance was evaluated using rolling-origin cross-validation, summarized by the mean absolute error (MAE) and root mean squared error (RMSE). Sensitivity analyses comparing alternative ARIMA specifications were also performed.

## 3. Results

### 3.1. Overall Mortality Trends

**Figure 1:**
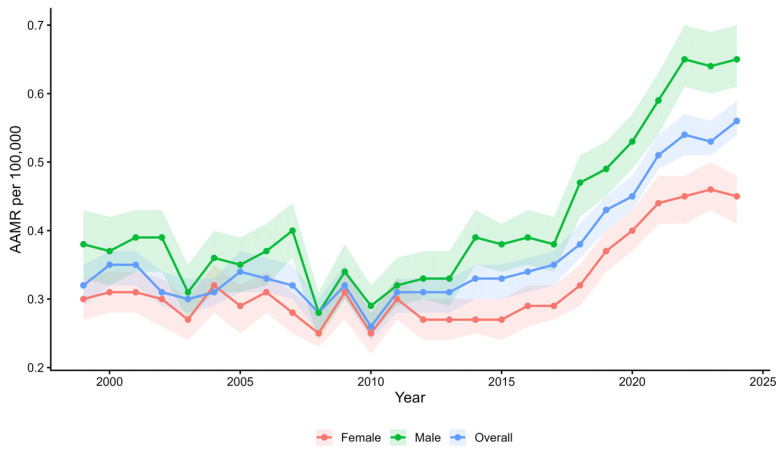
Overall and sex-stratified co-recorded pancreatic cancer and cerebrovascular disease mortality rates per 100,000, in the United States, 1999 to 2024. Shaded bands represent 95% confidence intervals.

From 1999 to 2024, there were 22,085 co-recorded pancreatic cancer and cerebrovascular disease deaths in the United States among adults aged 25 years and older. Annual deaths increased from 591 in 1999 to 1630 in 2024. The overall age-adjusted mortality rate (AAMR) increased from 0.32 per 100,000 in 1999 to 0.56 per 100,000 in 2024 (Figure 1). Annual death counts, population denominators, AAMRs, and 95% confidence intervals are presented in Table 1. Joinpoint regression identified one inflection point in 2013. AAMRs were stable from 1999 to 2013 (APC: −0.90; 95% CI: −2.33 to 0.19), followed by a significant increase from 2013 to 2024 (APC: 6.26; 95% CI: 5.11 to 8.25). Across the full study period, mortality increased significantly (AAPC: 2.19; 95% CI: 1.81 to 2.67). Complete segment-specific APC and full-period AAPC estimates for the overall population, and all demographic and geographic strata are presented in Table 2, Panel A.

### 3.2. Comparison with Single-Disease Mortality

To place the co-recorded mortality trend in context, we compared it with underlying-cause mortality from each condition individually. Pancreatic cancer underlying-cause deaths increased from 29,077 (AAMR, 16.41 per 100,000) in 1999 to 51,313 (AAMR, 17.53 per 100,000) in 2024. Cerebrovascular disease underlying-cause deaths were similar in 1999 and 2024 (166,968 vs. 166,437), although the corresponding AAMR decreased from 95.04 to 59.49 per 100,000. Over the same period, co-recorded pancreatic cancer and cerebrovascular disease deaths increased from 591 (AAMR, 0.32 per 100,000) to 1630 (AAMR, 0.56 per 100,000). Expressed as a ratio of co-recorded deaths to single-disease underlying-cause deaths, co-recorded deaths per 100 pancreatic cancer underlying-cause deaths rose from 2.03 in 1999 to 3.18 in 2024, and co-recorded deaths per 100 cerebrovascular disease underlying-cause deaths rose from 0.35 in 1999 to 0.98 in 2024. These contrasting trends provide context for the increase in co-recorded mortality but do not establish the mechanisms underlying that increase. The ratios describe a change in the relative frequency of co-recording and should not be interpreted as patient-level risks, incidence proportions, or case-fatality measures. Annual values for all three series are presented in Table 1.

### 3.3. Sex-Specific Trends

Mortality rates were consistently higher among males than females. From 1999 to 2024, the AAMR increased from 0.38 to 0.65 per 100,000 among males and from 0.30 to 0.45 per 100,000 among females. Overall, there were 10,999 male deaths and 11,086 female deaths. Among females, AAMRs declined from 1999 to 2015 (APC: −0.84; 95% CI: −1.74 to −0.07), then increased significantly from 2015 to 2024 (APC: 7.29; 95% CI: 5.81 to 9.37). Among males, AAMRs declined from 1999 to 2012 (APC: −1.54; 95% CI: −3.20 to −0.19), followed by a significant increase from 2012 to 2024 (APC: 6.64; 95% CI: 5.57 to 8.39). Full-period trends were significantly increasing for both females (AAPC: 2.01; 95% CI: 1.55 to 2.48) and males (AAPC: 2.31; 95% CI: 1.90 to 2.83).

### 3.4. Race and Ethnicity-Specific Trends

**Figure 2:**
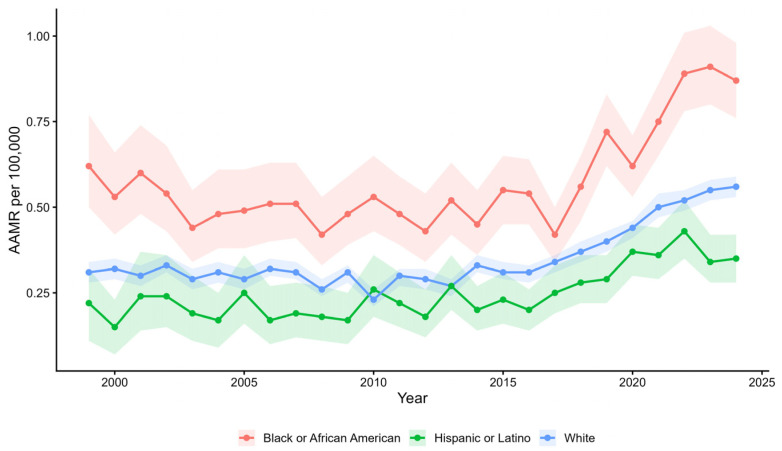
Co-recorded pancreatic cancer and cerebrovascular disease mortality rates per 100,000, stratified by race/ethnicity, in the United States, 1999 to 2024. Shaded bands represent 95% confidence intervals.

Race/ethnicity-stratified analyses showed persistent disparities (Figure 2). Non-Hispanic Black or African American individuals had the highest AAMRs throughout the study period, increasing from 0.62 per 100,000 in 1999 to 0.87 per 100,000 in 2024. Rates increased from 0.31 to 0.56 per 100,000 among non-Hispanic White individuals and from 0.22 to 0.35 per 100,000 among Hispanic or Latino individuals. Among non-Hispanic Black or African American individuals, AAMRs were stable from 1999 to 2014, then increased significantly from 2014 to 2024 (APC: 7.51; 95% CI: 5.12 to 12.52). Among non-Hispanic White individuals, AAMRs were stable from 1999 to 2013, then increased significantly from 2013 to 2024 (APC: 6.71; 95% CI: 5.25 to 8.63). Hispanic or Latino individuals had a significant full-period increase (AAPC: 2.12; 95% CI: 1.11 to 3.80); segment-level trends were not statistically significant, including a 2016–2022 segment with a large but imprecise point estimate (APC, 11.26; 95% CI, −1.40 to 24.39), reflecting the smaller number of deaths in this subgroup.

### 3.5. Regional Trends

**Figure 3:**
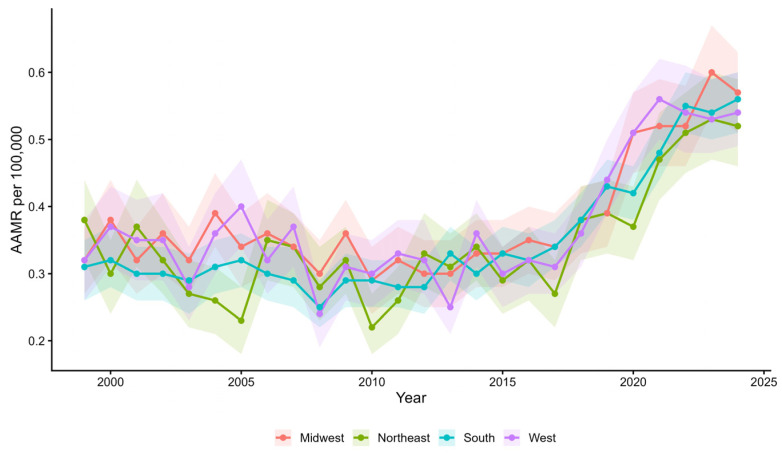
Co-recorded pancreatic cancer and cerebrovascular disease mortality rates per 100,000, stratified by Census region, in the United States, 1999 to 2024. Shaded bands represent 95% confidence intervals.

Mortality increased significantly across all Census regions over the full study period, with the highest 2024 AAMR observed in the Midwest at 0.57 per 100,000, followed by the South at 0.56, West at 0.54, and Northeast at 0.52 (Figure 3). The South accounted for the largest number of deaths overall, followed by the Midwest, West, and Northeast. The South had the largest full-period increase (AAPC: 2.62; 95% CI: 2.29 to 3.02), followed by the Northeast (AAPC: 2.26; 95% CI: 1.33 to 3.32), Midwest (AAPC: 2.20; 95% CI: 1.54 to 3.02), and West (AAPC: 1.75; 95% CI: 0.97 to 2.49). The West region’s 2017–2020 segment showed a large but statistically non-significant annual percent change (APC, 21.10; 95% CI, −0.34 to 27.46), illustrating that some segment-specific estimates in smaller strata are imprecise despite large point estimates.

### 3.6. Urbanization Trends

**Figure 4:**
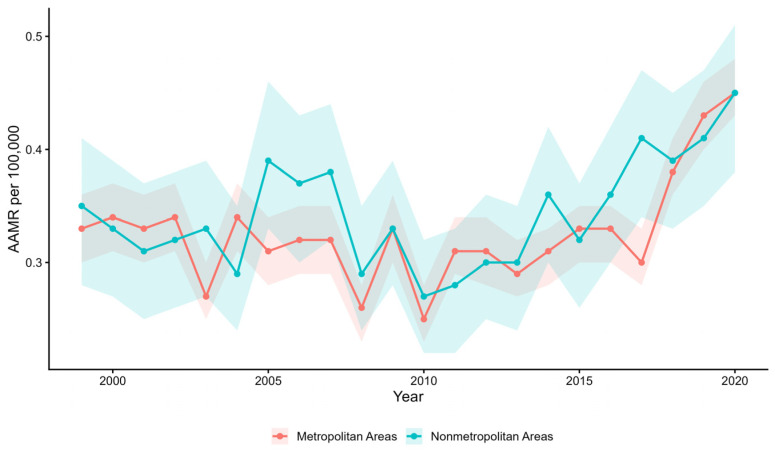
Co-recorded pancreatic cancer and cerebrovascular disease mortality rates per 100,000, stratified by urbanization, in the United States, 1999 to 2020. Shaded bands represent 95% confidence intervals.

Urbanization analyses were limited to 1999 through 2020. AAMRs increased from 0.33 to 0.45 per 100,000 in metropolitan areas and from 0.35 to 0.45 per 100,000 in nonmetropolitan areas (Figure 4). Overall, there were 13,012 deaths in metropolitan areas and 2946 deaths in nonmetropolitan areas during this period. Metropolitan AAMRs were stable from 1999 to 2017, followed by a significant increase from 2017 to 2020 (APC: 14.22; 95% CI: 6.63 to 25.68). Nonmetropolitan AAMRs increased significantly from 2010 to 2020 (APC: 4.95; 95% CI: 3.38 to 7.94). An earlier 1999–2007 nonmetropolitan segment showed a small, non-significant increase (APC, 1.68; 95% CI, −0.24 to 7.86), followed by a significant decline from 2007 to 2010 (APC, −9.30; 95% CI, −13.26 to −1.33) before the 2010–2020 increase reported above; this three-segment pattern should be interpreted cautiously given the smaller number of nonmetropolitan deaths relative to metropolitan areas. Full-period trends were significantly increasing in both metropolitan areas (AAPC: 2.08; 95% CI: 1.32 to 2.82) and nonmetropolitan areas (AAPC: 1.55; 95% CI: 1.02 to 2.39).

### 3.7. State-Level Variation

**Figure 5:**
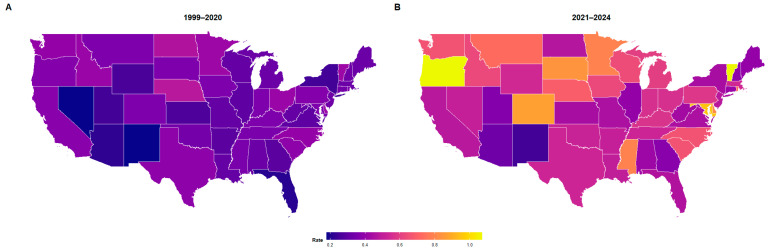
Co-recorded pancreatic cancer and cerebrovascular disease mortality rates per 100,000, stratified by state, comparing 1999–2020 (**A**) and 2021–2024 (**B**) in the United States. Both panels use a single shared color scale for direct visual comparison. Exact state-level death counts, AAMRs, and 95% confidence intervals are provided in Table 2, Panel B. Exact state-level death counts, AAMRs, and 95% confidence intervals are provided in Table 2.

State-level mortality varied substantially (Figure 5). State estimates were summarized separately for 1999–2020 and 2021–2024 because CDC WONDER provides two overlapping Multiple Cause of Death databases covering this period: Multiple Cause of Death, 1999–2020 and Multiple Cause of Death, 2018–2024, Single Race. To construct non-overlapping calendar periods, estimates for 1999–2020 were obtained from the former database and estimates for 2021–2024 from the latter. The earlier window pools 22 years of data, whereas the later window pools only four years and is therefore more susceptible to fluctuation from small numbers, particularly in low-population states. During 1999 to 2020, the highest AAMRs were observed in the District of Columbia at 0.51 per 100,000, Alaska at 0.50, Nebraska at 0.48, and North Dakota at 0.46. The lowest rates were observed in New Mexico and Nevada, both at 0.18 per 100,000, followed by Florida at 0.21 and Arizona at 0.22.

During 2021 to 2024, the highest AAMRs were observed in Oregon and Vermont, both at 1.07 per 100,000, followed by Maryland at 0.95, Colorado at 0.87, and South Dakota at 0.84. The lowest recent rates were observed in New Mexico at 0.25 per 100,000, Arizona at 0.33, Utah at 0.37, and Georgia at 0.38. These rankings should be interpreted cautiously because the estimates cover only four years and several were based on small numbers. For example, estimates for Alaska and the District of Columbia were based on 11 and 12 deaths, respectively, during 2021–2024. State-specific death counts, AAMRs, and 95% confidence intervals for 1999–2020 and 2021–2024 are presented in Table 2, Panel B.

### 3.8. Forecasted Mortality Trends

**Figure 6:**
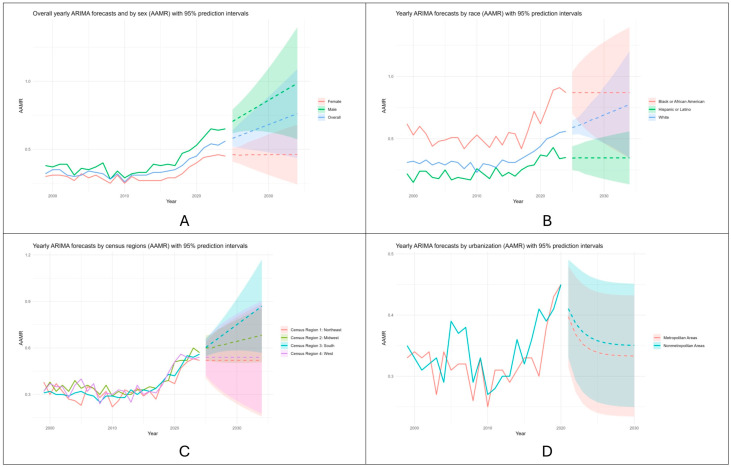
ARIMA-forecasted, exploratory age-adjusted mortality rates for co-recorded pancreatic cancer and cerebrovascular disease deaths in the United States, stratified by (**A**) sex, (**B**) race/ethnicity, (**C**) US Census region, and (**D**) urbanization status. Solid lines represent observed rates, and dashed lines represent projected rates; shaded areas indicate 95% prediction intervals. AAMR, age-adjusted mortality rate; ARIMA, autoregressive integrated moving average selected model orders, AIC, BIC, residual diagnostics, and rolling-origin validation metrics are presented in Table 2, Panel C. Forecasts should nevertheless be interpreted as exploratory because of the short annual time series.

ARIMA-based projections suggested that overall mortality will continue to rise modestly through 2034. The overall AAMR is projected to increase to 0.76 per 100,000 by 2034, with a wide 95% prediction interval of 0.43 to 1.09 that reflects the uncertainty inherent in extrapolating a 10-year horizon from a relatively short historical series (Figure 6).

Male mortality is projected to remain higher than female mortality, reaching 0.99 per 100,000 by 2034 compared with 0.46 per 100,000 among females. Race/ethnicity-specific forecasts suggest that non-Hispanic Black or African American individuals will continue to have the highest projected mortality burden, remaining near 0.87 per 100,000 through 2034, while non-Hispanic White mortality is projected to increase to 0.77 per 100,000. Regional forecasts suggest the highest projected 2034 AAMR in the South at 0.87 per 100,000, followed by the Midwest at 0.68, West at 0.54, and Northeast at 0.52. Urbanization forecasts through 2030 remained broadly stable, with projected AAMRs of 0.33 per 100,000 in metropolitan areas and 0.35 per 100,000 in nonmetropolitan areas. Given the short annual time series, these forecasts should be regarded as exploratory extrapolations rather than validated predictions. Selected ARIMA model orders and corresponding AIC and BIC values are presented in Table 2, Panel C.

## 4. Discussion

This national multiple-cause mortality analysis identified 22,085 deaths involving pancreatic cancer and cerebrovascular disease among US adults aged 25 years or older between 1999 and 2024. The age-adjusted mortality rate (AAMR) increased from 0.32 to 0.56 per 100,000, with an inflection after 2013 and an annual increase of 6.26% thereafter. Increases occurred in both sexes and all Census regions, while racial and geographic heterogeneity persisted. Males had higher AAMRs than females, and non-Hispanic Black or African American individuals experienced the highest mortality. These findings quantify deaths in which both conditions were recorded but do not establish causality or the proximate cause of death.

Although the observed increase is consistent with previous reports describing thromboembolic complications among patients with pancreatic cancer, the present ecological analysis cannot determine whether cerebrovascular disease was causally related to pancreatic cancer in individual patients. Population-based studies have shown increased short-term risks after cancer diagnosis, with pancreatic cancer among the malignancies carrying the greatest excess risk [3,4,5]. Among 838 patients receiving palliative chemotherapy, arterial thromboembolism occurred in 42 (5.0%) and was associated with shorter median survival (5.1 vs. 7.8 months) [10]. In the prospective BACAP cohort, 152 of 731 patients (20.79%) developed venous thromboembolism, independently associated with shorter overall survival (hazard ratio [HR], 2.02; 95% confidence interval [CI], 1.57–2.60) [11], supporting a systemic prothrombotic phenotype. These clinical cohort findings describe thromboembolic risk among patients with an established pancreatic cancer diagnosis and cannot be applied directly to the population-level, multiple-cause mortality data analysed here, which cannot distinguish clinically confirmed cancer-associated stroke from coincidental co-occurrence of the two conditions on a single death certificate.

Previous clinical studies have proposed several potential mechanisms, including cancer-associated hypercoagulability, disseminated intravascular coagulation, and nonbacterial thrombotic endocarditis. A cohort of 17 patients with pancreatic cancer and cerebral ischaemic events reported disseminated infarction patterns [12]. In a case–control study of 140 patients with active cancer and stroke and 140 matched controls, cancer was associated with multiple-territory infarctions, unidentified mechanisms and elevated D-dimer concentrations; pancreatic and lung cancers were over-represented among suspected cancer-associated strokes [13]. Similar patterns were reported in a multicentre study [14]. In the 268-patient OASIS-CANCER cohort, high pretreatment D-dimer concentrations were independently associated with poorer overall survival (adjusted HR, 2.19; 95% CI, 1.46–3.31) and 1-year survival (adjusted HR, 2.70; 95% CI, 1.68–4.35) [15]. In another study of 263 patients with active cancer and acute ischaemic stroke, cumulative 6-month rates of recurrent thromboembolism and recurrent ischaemic stroke were 37% and 16%, respectively [16]. While these clinical cohorts provide biological context, our population-level data cannot confirm specific stroke mechanisms or causal sequences.

Several factors likely drive the post-2013 acceleration. Pancreatic cancer incidence and mortality have increased globally [1,2], with SEER data showing increasing incidence across demographic groups [17]. Population ageing, increasing pancreatic cancer incidence, evolving treatment patterns, and changes in death certification or diagnostic coding may all have contributed. Previous modelling studies have projected pancreatic cancer to become the second leading cause of cancer-related death in the United States by 2030 [18]. This study cannot separate the effects of incidence, survival, treatment, stroke trends, coding or death certification.

Racial disparities were pronounced. In 2024, AAMRs were 0.87 per 100,000 among non-Hispanic Black or African American individuals, 0.56 among non-Hispanic White individuals and 0.35 among Hispanic or Latino individuals. National data similarly showed higher pancreatic cancer incidence (24.7 vs. 19.4 per 100,000; incidence rate ratio, 1.28; 95% CI, 1.26–1.29) and mortality (23.3 vs. 18.4 per 100,000; mortality rate ratio, 1.27; 95% CI, 1.26–1.28) among Black than White individuals [19]. Black patients with locoregional disease were less likely to undergo surgical evaluation (adjusted odds ratio, 0.57; 95% CI, 0.42–0.77) [20], while African American race independently predicted higher mortality [21]. In REGARDS, traditional vascular and socioeconomic factors explained approximately half of excess stroke risk among Black adults aged 45–65 years [22]. The observed disparity may reflect differences in cancer burden, vascular risk, access and structural disadvantage. Sex-based disparities were also evident, with male AAMRs exceeding female AAMRs throughout the study period and reaching 0.65 versus 0.45 per 100,000 in 2024. The significant increase began in 2012 among males and in 2015 among females. The present data cannot determine whether this disparity reflects sex differences in pancreatic cancer burden, cerebrovascular disease burden, the co-occurrence of the two conditions, or death certification practices.

Mortality increased across all Census regions. The South had the largest full-period increase, whereas the Midwest had the highest 2024 AAMR. Metropolitan and nonmetropolitan rates both reached 0.45 per 100,000 by 2020. Rural–urban differences in cancer incidence vary by cancer type, geography and modifiable risk factors [23]. State estimates may reflect access, coding and small numbers; comparisons between 1999–2020 and 2021–2024 require caution because the latter interval was shorter.

ARIMA modelling projected an overall AAMR of 0.76 per 100,000 by 2034, with higher male mortality and persistent racial disparity. These projections extrapolate historical patterns and exclude future changes in population composition, treatment, thromboprophylaxis, stroke prevention or coding; widening prediction intervals limit precision.

Strengths include national coverage over 26 years, inclusion of underlying and contributing causes, and consistent subgroup and forecasting analyses. Limitations include death certificate misclassification: historical validation found the recorded underlying cancer site accurate for approximately 65% of cancer deaths [24], while an autopsy-based study reported 48% overall agreement for underlying cause of death and 81% agreement for cancer [25]. Requiring both conditions may underestimate coexistence, while co-recording does not establish temporal sequence or causality. AAMRs represent population mortality burden, not cerebrovascular risk among patients with pancreatic cancer, and this ecological design cannot separate the effects of pancreatic cancer burden, population aging, cerebrovascular disease prevalence, diagnostic coding, and death certificate documentation on the observed trend. ICD-10 codes I60–I69 combine ischemic stroke, hemorrhagic stroke, and other cerebrovascular diseases, preventing subtype-specific interpretation. The data also lack cancer stage, histology, treatment, anticoagulant exposure, vascular risk factors, performance status, and socioeconomic measures. The study period includes the COVID-19 pandemic, during which disrupted diagnostic evaluation and care delivery, competing mortality, and changes in death certification practices may have affected the observed trends. The ARIMA forecasts were generated using the auto.arima algorithm with model selection based on information criteria and were evaluated using residual diagnostics and rolling-origin cross-validation. Nevertheless, the forecasts remain exploratory because they are based on only 26 annual observations and cannot account for future changes in treatment, prevention, population structure, or death certification practices. Urbanization data ended in 2020, and forecasts remain model-dependent.

In conclusion, US co-recorded pancreatic cancer and cerebrovascular disease mortality has risen substantially since 2013 and remains disproportionately high among males and non-Hispanic Black or African American individuals. These findings arise from an ecological, multiple-cause-of-death analysis that cannot establish causality, temporal sequence, or cerebrovascular risk at the individual patient level. Linked patient-level datasets incorporating cancer stage, treatment, cerebrovascular subtype, vascular risk factors, and socioeconomic characteristics are needed to clarify the factors underlying this co-occurrence.

## Figures and Tables

**Table 1:** Annual Co-Recorded Mortality and Disease-Specific Comparator Ratios, United States, 1999–2024.

Year	Co-Recorded Deaths, *n*	Population	AAMR per 100,000	95% CI	Pancreatic Cancer Deaths, n	Ratio per 100 Pancreatic Cancer Deaths	Cerebrovascular Deaths, n	Ratio per 100 Cerebrovascular Deaths
1999	591	180,408,769	0.32	0.29, 0.35	29,077	2.03	166,968	0.35
2000	622	181,984,640	0.35	0.32, 0.37	29,322	2.12	167,213	0.37
2001	604	184,305,128	0.35	0.32, 0.37	29,797	2.03	163,096	0.37
2002	611	186,208,028	0.31	0.29, 0.34	30,255	2.02	162,237	0.38
2003	558	188,090,429	0.3	0.28, 0.33	30,766	1.81	157,250	0.35
2004	626	190,205,384	0.31	0.29, 0.33	31,760	1.97	149,613	0.42
2005	619	192,551,384	0.34	0.31, 0.37	32,754	1.89	143,096	0.43
2006	635	195,019,359	0.33	0.31, 0.36	33,446	1.9	136,617	0.46
2007	640	197,403,777	0.32	0.30, 0.35	34,110	1.88	135,484	0.47
2008	566	199,795,090	0.28	0.25, 0.30	35,225	1.61	133,653	0.42
2009	682	202,107,016	0.32	0.30, 0.35	35,617	1.91	128,377	0.53
2010	580	203,891,983	0.26	0.24, 0.28	36,877	1.57	129,014	0.45
2011	657	206,592,936	0.31	0.28, 0.33	37,339	1.76	128,481	0.51
2012	666	208,826,037	0.31	0.28, 0.33	38,789	1.72	128,116	0.52
2013	686	211,085,314	0.31	0.28, 0.33	38,984	1.76	128,586	0.53
2014	772	213,809,280	0.33	0.30, 0.35	40,403	1.91	132,708	0.58
2015	790	216,553,817	0.33	0.30, 0.35	41,601	1.9	139,934	0.56
2016	810	218,641,417	0.34	0.31, 0.36	42,751	1.89	141,719	0.57
2017	860	221,447,331	0.35	0.32, 0.37	44,007	1.95	145,963	0.59
2018	981	223,311,190	0.38	0.36, 0.41	44,903	2.18	147,432	0.67
2019	1145	224,981,167	0.43	0.40, 0.45	45,877	2.5	149,603	0.77
2020	1257	226,635,013	0.45	0.43, 0.48	46,765	2.69	159,839	0.79
2021	1393	228,238,412	0.51	0.49, 0.54	47,897	2.91	162,459	0.86
2022	1536	229,508,599	0.54	0.51, 0.57	48,316	3.18	164,998	0.93
2023	1568	231,529,762	0.53	0.51, 0.56	49,441	3.17	162,238	0.97
2024	1630	235,615,087	0.56	0.54, 0.59	51,313	3.18	166,437	0.98

AAMR = age-adjusted mortality rate; CI = confidence interval. Co-recorded deaths indicate death certificates listing both pancreatic cancer and cerebrovascular disease. Ratio columns show co-recorded deaths per 100 underlying-cause deaths for each disease.

**Table 2:** Joinpoint Results, State-Level Mortality Estimates, and ARIMA Model Diagnostics. Panel (**A**). Joinpoint trends. Panel (**B**). State-level mortality. Panel (**C**). ARIMA diagnostics and validation.

(**A**)											
**Stratification**		**Subgroup**	**Segment Years**		**APC, %**	**APC 95% CI**		***p* Value**	**Full-Period AAPC, %**	**AAPC 95% CI**	
Overall		Overall	1999–2013		−0.90	−2.33, 0.19		0.096	2.19	1.81, 2.67	
Overall		Overall	2013–2024		6.26	5.11, 8.25		<0.001			
Sex		Male	1999–2012		−1.54	−3.20, −0.19		0.023	2.31	1.90, 2.83	
Sex		Male	2012–2024		6.64	5.57–8.39		<0.001			
Sex		Female	1999–2015		−0.84	−1.74, −0.07		0.031	2.01	1.55, 2.48	
Sex		Female	2015–2024		7.29	5.81, 9.37		<0.001			
Race/ethnicity		White	1999–2013		−0.94	−2.14, 0.05		0.062	2.36	1.87, 2.86	
Race/ethnicity		White	2013–2024		6.71	5.25, 8.63		<0.001			
Race/ethnicity		Black or African American	1999–2014		−1.35	−4.07, 0.48		0.145	2.10	1.34, 3.05	
Race/ethnicity		Black or African American	2014–2024		7.51	5.12, 12.52		<0.001			
Race/ethnicity		Hispanic or Latino	1999–2016		0.53	−3.22, 8.41		0.559	2.12	1.11, 3.80	
Race/ethnicity		Hispanic or Latino	2016–2022		11.26	−1.40, 24.39		0.064			
Race/ethnicity		Hispanic or Latino	2022–2024		−9.81	−20.58, 6.31		0.169			
Census region		Northeast	1999–2016		−0.64	−3.56, 1.04		0.409	2.26	1.33, 3.32	
Census region		Northeast	2016–2024		8.72	4.90, 19.88		<0.001			
Census region		Midwest	1999–2015		−0.76	−3.03, 0.69		0.257	2.20	1.54, 3.02	
Census region		Midwest	2015–2024		7.69	5.44, 15.05		<0.001			
Census region		South	1999–2014		−0.30	−1.34, 0.54		0.467	2.62	2.29, 3.02	
Census region		South	2014–2024		7.16	6.07, 8.81		<0.001			
Census region		West	1999–2017		−0.88	−2.30, 0.39		0.118	1.75	0.97, 2.49	
Census region		West	2017–2020		21.10	−0.34, 27.46		0.054			
Census region		West	2020–2024		0.47	−8.65, 7.22		0.998			
Urbanization		Metropolitan	1999–2017		0.18	−0.91, 1.13		0.718	2.08	1.32, 2.82	
Urbanization		Metropolitan	2017–2020		14.22	6.63, 25.68		<0.001			
Urbanization		Nonmetropolitan	1999–2007		1.68	−0.24–7.86		0.071	1.55	1.02, 2.39	
Urbanization		Nonmetropolitan	2007–2010		−9.30	−13.26, −1.33		0.033			
Urbanization		Nonmetropolitan	2010–2020		4.95	3.38, 7.94		0.014			
(**B**)											
**State**		**Deaths, 1999–2020**	**AAMR, 1999–2020**		**95% CI, 1999–2020**	**Deaths, 2021–2024**		**AAMR, 2021–2024**		**95% CI, 2021–2024**	
Alabama		242	0.32		0.28, 0.36	73		0.42		0.32, 0.53	
Alaska		33	0.5		0.34, 0.72	11		0.51		0.25, 1.00	
Arizona		207	0.22		0.19, 0.25	90		0.33		0.26, 0.41	
Arkansas		143	0.29		0.24, 0.34	53		0.51		0.38, 0.68	
California		1952	0.38		0.37, 0.40	594		0.48		0.44, 0.52	
Colorado		234	0.36		0.32, 0.41	159		0.87		0.74, 1.02	
Connecticut		212	0.34		0.29, 0.39	55		0.41		0.31, 0.54	
Delaware		60	0.42		0.32, 0.54	33		0.81		0.55, 1.18	
District of Columbia		41	0.51		0.36, 0.69	12		0.69		0.36, 1.22	
Florida		857	0.21		0.19, 0.22	448		0.46		0.42, 0.51	
Georgia		348	0.29		0.26, 0.32	127		0.38		0.32, 0.46	
Hawaii		99	0.44		0.35, 0.53	32		0.55		0.37, 0.81	
Idaho		91	0.41		0.33, 0.50	38		0.63		0.44, 0.89	
Illinois		587	0.3		0.28, 0.33	176		0.4		0.34, 0.47	
Indiana		362	0.36		0.32, 0.39	126		0.56		0.46, 0.67	
Iowa		191	0.36		0.31, 0.41	72		0.63		0.49, 0.81	
Kansas		121	0.27		0.22, 0.32	42		0.44		0.32, 0.60	
Kentucky		249	0.36		0.31, 0.40	81		0.57		0.45, 0.71	
Louisiana		205	0.31		0.27, 0.36	79		0.5		0.39, 0.63	
Maine		81	0.32		0.25, 0.40	24		0.4		0.25, 0.64	
Maryland		320	0.36		0.32, 0.40	194		0.95		0.82, 1.10	
Massachusetts		341	0.32		0.29, 0.36	134		0.54		0.45, 0.65	
Michigan		530	0.32		0.29, 0.35	216		0.6		0.52, 0.69	
Minnesota		357	0.42		0.38, 0.47	156		0.79		0.67, 0.93	
(**C**)											
**Series**	**Selected Model**	**AIC**	**BIC**	**Coefficients, Estimate (SE)**	**Ljung–Box Statistic**	**Ljung–Box *p* Value**	**Rolling-origin MAE**	**Rolling-origin RMSE**	**Naïve RMSE**	**Alternative Model**	**Alternative-Model RMSE**
Overall	ARIMA (0, 2, 1)	−95.2	−92.85	ma1 = −0.849 (0.11)	6.14	0.803	0.028	0.035	0.03	ARIMA (0, 2, 1)	0.038
Male	ARIMA (0, 2, 2)	−75.39	−71.86	ma1 = −1.294 (0.253); ma2 = 0.482 (0.256)	9.87	0.452	0.044	0.057	0.047	ARIMA (0, 2, 2)	0.057
Female	ARIMA (3, 1, 0)	−104.49	−99.62	ar1 = −0.347 (0.18); ar2 = 0.418 (0.169); ar3 = 0.332 (0.177)	5.36	0.866	0.03	0.04	0.031	ARIMA (0, 1, 2)	0.04
White	ARIMA (2, 2, 0)	−95.97	−92.44	ar1 = −1.283 (0.139); ar2 = −0.661 (0.136)	5.11	0.884	0.033	0.047	0.038	ARIMA (2, 2, 0)	0.046
Black or African American	ARIMA (0, 1, 0)	−50.52	−49.3	—	18.76	0.043	0.08	0.101	0.084	ARIMA (0, 1, 0)	0.103
Hispanic or Latino	ARIMA (1, 1, 0)	−76.67	−74.23	ar1 = −0.498 (0.173)	8.46	0.584	0.044	0.05	0.055	ARIMA (1, 1, 0)	0.05
Census Region 1: Northeast	ARIMA (0, 1, 0)	−69.04	−67.82	—	13.09	0.219	0.057	0.073	0.057	ARIMA (0, 1, 0)	0.076
Census Region 2: Midwest	ARIMA (1, 1, 0)	−84.89	−81.24	ar1 = −0.522 (0.171); drift = 0.01 (0.005)	12.38	0.26	0.043	0.061	0.046	ARIMA (0, 1, 2)	0.06
Census Region 3: South	ARIMA (0, 2, 2)	−97.07	−93.54	ma1 = −1.234 (0.25); ma2 = 0.459 (0.245)	8.09	0.62	0.027	0.035	0.031	ARIMA (0, 2, 1)	0.035
Census Region 4: West	ARIMA (0, 1, 0)	−68.98	−67.76	—	31.93	0	0.049	0.073	0.059	ARIMA (1, 1, 0)	0.073
Metropolitan Areas	ARIMA (1, 0, 0)	−72.67	−69.39	ar1 = 0.552 (0.215); intercept = 0.333 (0.019)	11.81	0.298	0.031	0.049	0.045	ARIMA (1, 0, 0)	0.049
Nonmetropolitan Areas	ARIMA (1, 0, 0)	−73.8	−70.53	ar1 = 0.605 (0.19); intercept = 0.35 (0.02)	5.79	0.833	0.039	0.045	0.044	ARIMA (1, 0, 0)	0.045

APC = annual percentage change; AAPC = average annual percentage change; CI = confidence interval. Joinpoint regression identified significant changes in temporal trends. Statistical significance was defined as *p* < 0.05. AAMR = age-adjusted mortality rate. Estimates are shown separately for 1999–2020 and 2021–2024. ARIMA = autoregressive integrated moving average; AIC = Akaike Information Criterion; AICc = corrected Akaike Information Criterion; BIC = Bayesian Information Criterion; MAE = mean absolute error; RMSE = root mean squared error; SE = standard error.

## Data Availability

The data are publicly available through the CDC WONDER Multiple Cause of Death database. The query parameters used in this study are described in the Section 2.

## References

[1] Huang J., Lok V., Ngai C.H., Zhang L., Yuan J., Lao X.Q., Ng K., Chong C., Zheng Z.-J., Wong M.C. (2021). Worldwide burden of, risk factors for, and trends in pancreatic cancer. Gastroenterology.

[2] Cronin K.A., Scott S., Firth A.U., Sung H., Henley S.J., Sherman R.L., Siegel R.L., Anderson R.N., Kohler B.A., Benard V.B. (2022). Annual report to the nation on the status of cancer, part 1: National cancer statistics. Cancer.

[3] Navi B.B., Reiner A.S., Kamel H., Iadecola C., Elkind M.S.V., Panageas K.S., DeAngelis L.M. (2015). Association between incident cancer and subsequent stroke. Ann. Neurol..

[4] Chan P.-C., Chang W.-L., Hsu M.-H., Yeh C.-H., Muo C.-H., Chang K.-S., Hsu C.Y., Wu B.-T., Lai C.-H., Lee C.-H. (2018). Higher stroke incidence in the patients with pancreatic cancer: A nation-based cohort study in Taiwan. Medicine.

[5] Navi B.B., Reiner A.S., Kamel H., Iadecola C., Okin P.M., Elkind M.S., Panageas K.S., DeAngelis L.M. (2017). Risk of arterial thromboembolism in patients with cancer. J. Am. Coll. Cardiol..

[6] Centers for Disease Control and Prevention CDC WONDER [Internet]. Atlanta (GA): Centers for Disease Control and Prevention. https://wonder.cdc.gov/.

[7] von Elm E., Altman D.G., Egger M., Pocock S.J., Gøtzsche P.C., Vandenbroucke J.P., STROBE Initiative (2007). The Strengthening the Reporting of Observational Studies in Epidemiology (STROBE) statement: Guidelines for reporting observational studies. Lancet.

[8] Klein R.J., Schoenborn C.A. (2001). Age Adjustment Using the 2000 Projected U.S. Population; Healthy People 2010 Stat Notes.

[9] Hyndman R.J., Khandakar Y. (2008). Automatic time series forecasting: The forecast package for R. J. Stat. Softw..

[10] Hung Y.S., Chen J.S., Chen Y.Y., Lu C.H., Chang P.H., Chou W.C. (2018). Incidence, risk factors, and outcomes of arterial thromboembolism in patients with pancreatic cancer following palliative chemotherapy. Cancers.

[11] Frere C., Bournet B., Gourgou S., Fraisse J., Canivet C., Connors J.M., Buscail L., Farge D., BACAP Consortium (2020). Incidence of venous thromboembolism in patients with newly diagnosed pancreatic cancer and factors associated with outcomes. Gastroenterology.

[12] Bonnerot M., Humbertjean L., Mione G., Lacour J.C., Derelle A.L., Sanchez J.C., Riou-Comte N., Richard S. (2016). Cerebral ischemic events in patients with pancreatic cancer: A retrospective cohort study of 17 patients and a literature review. Medicine.

[13] Schwarzbach C.J., Schaefer A., Ebert A., Held V., Bolognese M., Kablau M., Hennerici M.G., Fatar M. (2012). Stroke and cancer: The importance of cancer-associated hypercoagulation as a possible stroke etiology. Stroke.

[14] Kim S.G., Hong J.M., Kim H.Y., Lee J., Chung P.W., Park K.Y., Kim G.M., Lee K.H., Chung C.S., Bang O.Y. (2010). Ischemic stroke in cancer patients with and without conventional mechanisms: A multicenter study in Korea. Stroke.

[15] Lee M.J., Chung J.-W., Ahn M.-J., Kim S., Seok J.M., Jang H.M., Kim G.-M., Chung C.-S., Lee K.H., Bang O.Y. (2017). Hypercoagulability and mortality of patients with stroke and active cancer: The OASIS-CANCER study. J. Stroke.

[16] Navi B.B., Singer S., Merkler A.E., Cheng N.T., Stone J.B., Kamel H., Iadecola C., Elkind M.S., DeAngelis L.M. (2014). Recurrent thromboembolic events after ischemic stroke in patients with cancer. Neurology.

[17] Gordon-Dseagu V.L., Devesa S.S., Goggins M., Stolzenberg-Solomon R.Z. (2018). Pancreatic cancer incidence trends: Evidence from the Surveillance, Epidemiology and End Results (SEER) population-based data. Int. J. Epidemiol..

[18] Rahib L., Smith B.D., Aizenberg R., Rosenzweig A.B., Fleshman J.M., Matrisian L.M. (2014). Projecting cancer incidence and deaths to 2030: The unexpected burden of thyroid, liver, and pancreas cancers in the United States. Cancer Res..

[19] Tavakkoli A., Singal A.G., Waljee A.K., Elmunzer B.J., Pruitt S.L., McKey T., Rubenstein J.H., Scheiman J.M., Murphy C.C. (2020). Racial disparities and trends in pancreatic cancer incidence and mortality in the United States. Clin. Gastroenterol. Hepatol..

[20] Riall T.S., Townsend C.M., Kuo Y.F., Freeman J.L., Goodwin J.S. (2010). Dissecting racial disparities in the treatment of patients with locoregional pancreatic cancer: A 2-step process. Cancer.

[21] Wray C.J., Castro-Echeverry E., Silberfein E.J., Ko T.C., Kao L.S. (2012). A multi-institutional study of pancreatic cancer in Harris County, Texas: Race predicts treatment and survival. Ann. Surg. Oncol..

[22] Howard G., Cushman M., Kissela B.M., Kleindorfer D.O., McClure L.A., Safford M.M., Rhodes J.D., Soliman E.Z., Moy C.S., Judd S.E. (2011). Traditional risk factors as the underlying cause of racial disparities in stroke: Lessons from the half-full (empty?) glass. Stroke.

[23] Zahnd W.E., James A.S., Jenkins W.D., Izadi S.R., Fogleman A.J., Steward D.E., Colditz G.A., Brard L. (2018). Rural-urban differences in cancer incidence and trends in the United States. Cancer Epidemiol. Biomark. Prev..

[24] Percy C., Stanek E., Gloeckler L. (1981). Accuracy of cancer death certificates and its effect on cancer mortality statistics. Am. J. Public Health.

[25] Mieno M.N., Tanaka N., Arai T., Kawahara T., Kuchiba A., Ishikawa S., Sawabe M. (2016). Accuracy of death certificates and assessment of factors for misclassification of underlying cause of death. J. Epidemiol..

